# Ingested sharp foreign body presented as chronic esophageal stricture and inflammatory mediastinal mass for 113 weeks: Case report

**DOI:** 10.1016/j.amsu.2019.07.028

**Published:** 2019-08-01

**Authors:** Nour A. Tashtush, Ziad A. Bataineh, Dawood H. Yusef, Thekraiat M. Al Quran, Liqa A. Rousan, Ruba Khasawneh, Abdelwahab J. Aleshawi, Eyad M. Altamimi

**Affiliations:** a Department Pediatrics and Neonatology, Faculty of Medicine, Jordan University of Science & Technology, Irbid, 22110, Jordan; b Department of General Surgery and Urology, Faculty of Medicine, Jordan University of Science & Technology, Irbid, 22110, Jordan; c Department of Public Health and Community Medicine, Faculty of Medicine, Jordan University of Science & Technology, Irbid, 22110, Jordan; d Department of Diagnostic and Interventional Radiology and Nuclear Medicine, Faculty of Medicine, Jordan University of Science & Technology, Irbid, 22110, Jordan

**Keywords:** Aluminum can tab, Mediastinitis, Chronic esophageal foreign body

## Abstract

**Introduction:**

Impacted foreign bodies in the esophagus have the potential to cause serious complications. Ingested sharp objects carry the risk of acute complications as: perforation, acute mediastinitis, and acute bleeding. Rarely, such foreign bodies might migrate through the esophageal wall and present as chronic esophageal foreign body.

**Case presentation:**

We present a case of a 36-month-old girl presented with solid food dysphagia and regurgitation proved to be secondary to esophageal stricture after 26 months of accidental ingestion of aluminum can tab which has migrated through the wall of the upper esophagus into the mediastinum. After two trials of endoscopic treatment; she underwent thoracotomy and partial esophagectomy. Multiple trials of dilation and Mitomycin C injection were followed because of re-stricture.

**Conclusion:**

Foreign body impaction or secondary stricture needs to be considered in the differential diagnosis of children presenting with new onset dysphagia and regurgitation. Metallic Foreign body might be even radiolucent. Practitioners should keep a high index of suspicion for a retained esophageal FB in the child with gastrointestinal or respiratory symptoms that do not respond to standard therapy.

## Highlights

• Foreign body impaction needs to be considered in the differential diagnosis of dysphagia and regurgitation.
• Metallic FBs might be even radiolucent, and X-ray might not be enough to exclude FB ingestion.
• Impacted FB in the esophagus may remain asymptomatic for some time and become symptomatic only when complications develop.
• Chronic esophageal foreign body can lead to complications that may enforce the physicians to go for aggressive surgeries.

## 1 Introduction

Foreign body (FB) ingestion is an everyday occurrence and a common emergency presentation in the pediatric population. Most of the FBs travel through the gastrointestinal tract without any complications. The natural narrowing areas through the gut serve as places for FB impaction [1]. Impacted FBs in the esophagus have the potential to cause serious complications, apart from significant distress to the patient and the family [1]. Esophagoscopy offers a safe and a widely available intervention for retrieval of FBs in the esophagus. Sometimes it is difficult to differentiate chronic esophageal foreign body (CEFB) ingestion from those patients with an upper respiratory tract infection or asthma or esophageal stenosis [2,3]. Ingested sharp objects carry the risk of perforating the esophagus leading to acute mediastinitis or acute bleeding, or rarely leading to abscess or fistula formation [4,5]. We present a case of a 36-month-old girl who presented with solid food dysphagia and regurgitation secondary to esophageal stricture after 26 months of accidental ingestion of an aluminum can tab that has migrated through the wall of the upper esophagus as a technical perforation.

This case study was performed and is being reported in line with the SCARE criteria [6].

## 2 Case presentation

Thirty-six-month-old girl; not known to have any medical or congenital illness; presented to our center complaining of persistent solid food dysphagia and regurgitation since the age of 10 months. However, her symptoms worsened over the last three months. The parents deny any history of recurrent choking or aspiration. She had no atopy, and her mother could not link her symptoms to any specific foods. Her drug, family and psychosocial history were irrelevant. Her examination revealed a normal looking child except for growth parameters which were on 5th percentile.

**Fig. 1:**
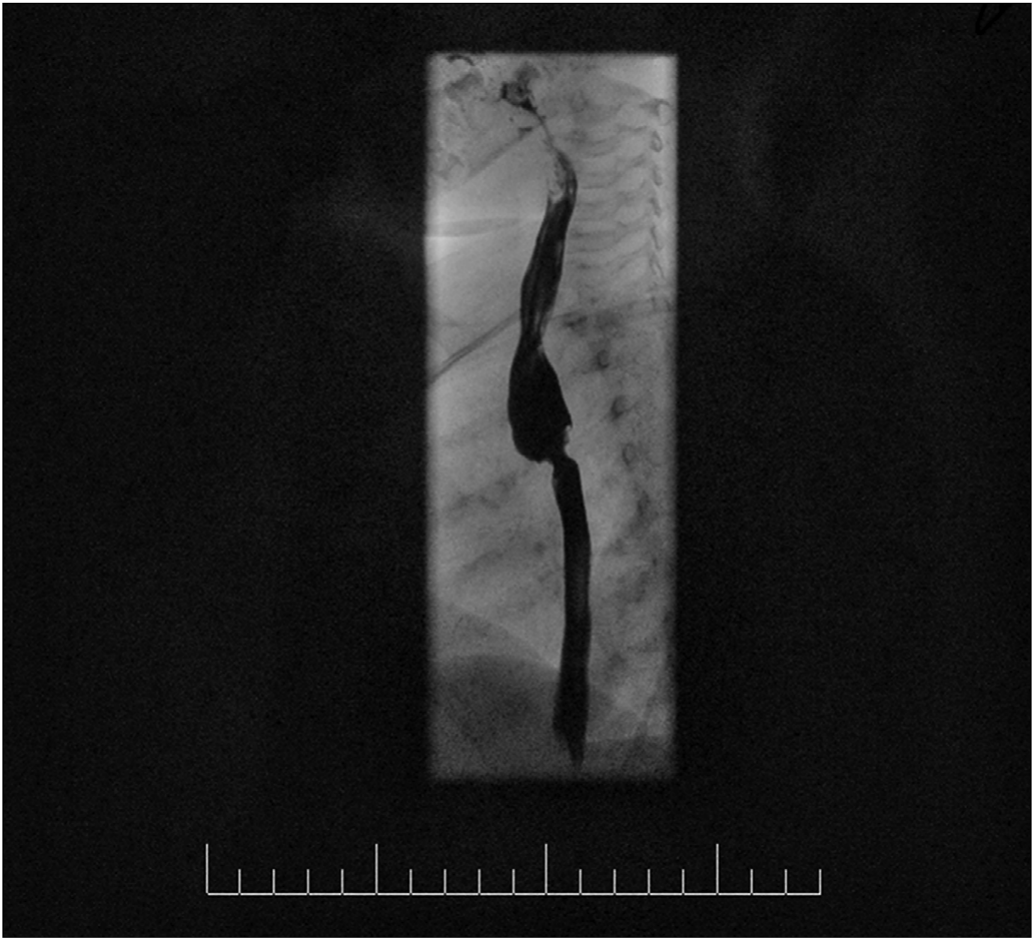
Non-ionized contrast swallow shows a short-narrowed segment in the midesophagus with pre-stenotic dilatation. There was no active leak of contrast during the study.

Her basic laboratory investigations were normal. Chest X-Ray was normal. She underwent diagnostic upper endoscopy by consultant pediatrics gastroenterologist (EA) and revealed a stricture at the upper esophagus with dilated pre-stenotic pouch filled with food. A flexible infant scope (Olympus ® 4.9 mm) could not pass through the stricture. Barium swallow was performed and delineated the anatomy of the stricture at the upper esophagus. Fig. 1.

**Fig. 2:**
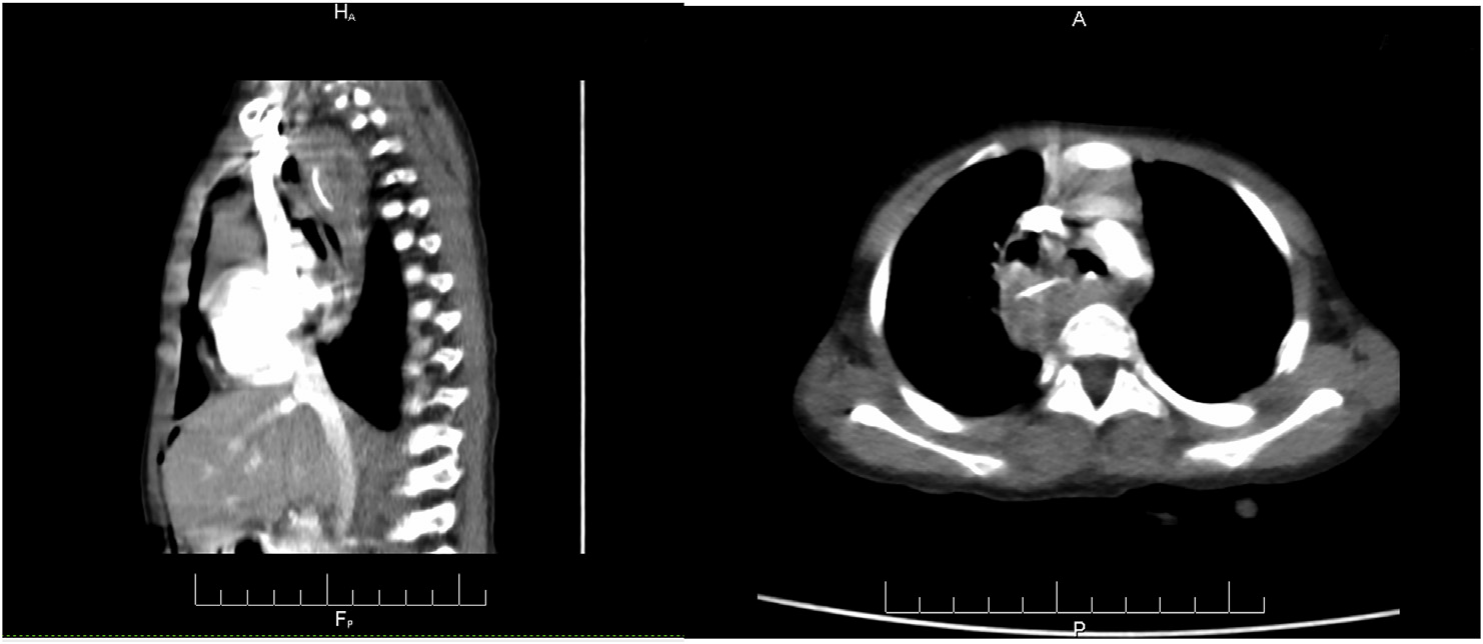
Enhanced chest CT scan axial and sagittal images show a linear dense object (arrow) in the middle mediastinum on the right side with surrounding soft tissue inflammatory phlegmon and multiple pockets of collections, causing narrowing the midesophagus.

Trial of antegrade esophageal dilatation (using flexible and rigid scopes) under general anesthesia failed at the first time, repeated four weeks later after gastrostomy and retrograde approach also failed by pediatrics surgeon (ZB) and (EA). Due to the fixed stricture and posterior outpouching, chest computed tomography (CT) scan with IV contrast was carried out. and revealed a circumferential soft tissue thickening involving the upper thoracic esophagus more pronounced at its right posterior lateral wall containing multiple rim-enhancing collections; the largest of which measures about 1.1 × 0.6 cm with linear hyperdense material measuring about 1.5 cm causing complete obstruction of the esophagus at this level and compression effect on the trachea which was displaced to the left side. Fig. 2.

The patient underwent thoracotomy by the pediatrics surgeon (ZB)_. An aluminum can tab with reactionary inflammatory mass around it were obstructing the esophagus and found to erode the esophageal wall with intramural elements. Removal of the foreign body along with the mass, partial esophagectomy with primary anastomosis were performed due to relatively wide esophageal perforation with in the inflammatory mass. No postoperative complications were detected. Fig. 3 Video 1.

**Fig. 3:**
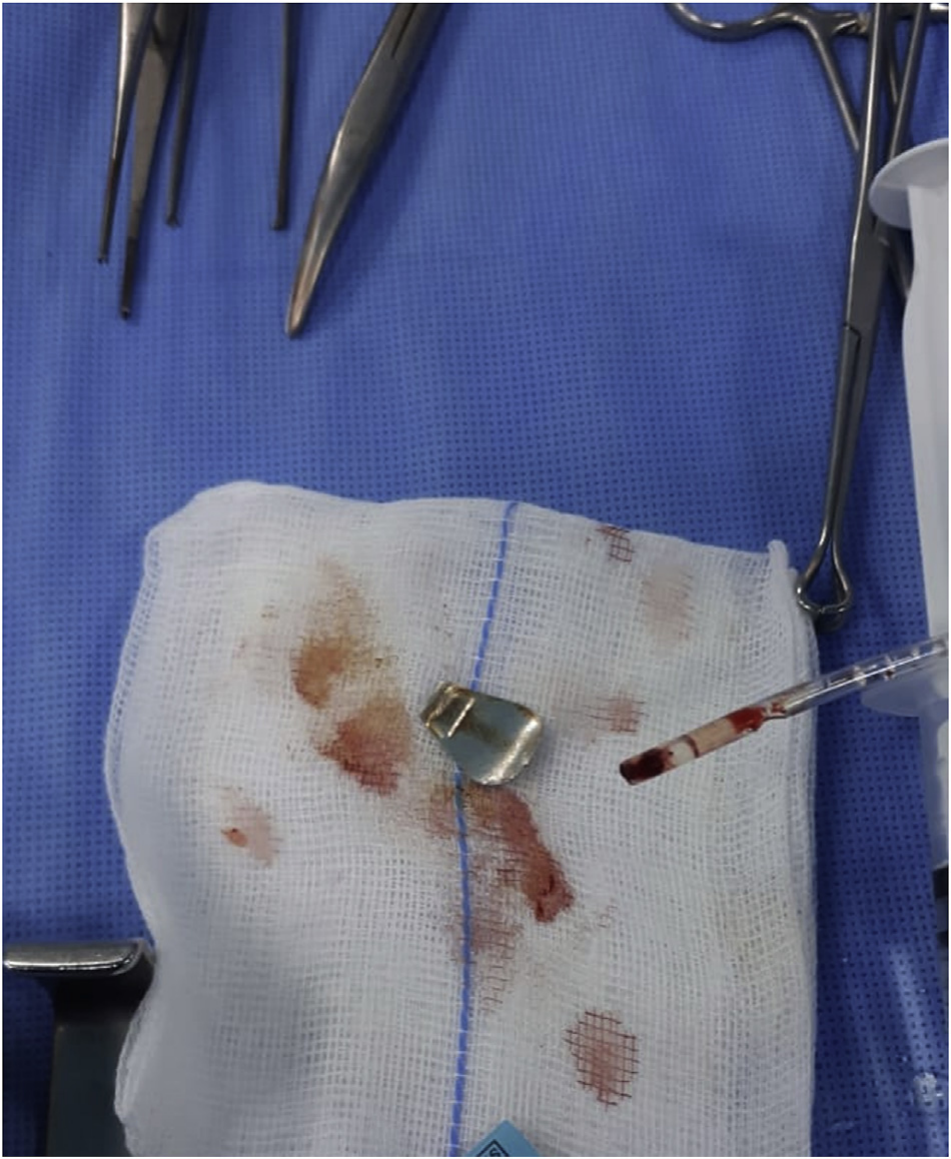
The aluminum can tab.

Supplementary video related to this article can be found at https://doi.org/10.1016/j.amsu.2019.07.028.

The following is the supplementary data related to this article:

video This video demonstrating the retrieving of the can tab. video

Retrospectively, the mother recalled a history of chocking episode and vomiting at the age of 10 months, which was not investigated at that time. After recovery, she was able to tolerate oral intake.

She presented two weeks later with dysphagia and regurgitation. Her upper endoscopy revealed re-stricture formation at the esophagectomy site. She underwent multiple sessions of esophageal dilatation using buggies up to 11mm by (EA). Due to the re-stricture the decision was to apply Mitomycin to reduce the need to re-dilatation. Mitomycin C was prepared immediately before application at a concentration of 0.4 mg/ml and applied at the site of dilatation. The last dilatation session was four months ago (6 months postoperatively). The parents were taught about the risk of FBs ingestion and about the importance of notice any episodes of chocking and they were adherent. The patient is doing well; she gained weight and is able to tolerate solid food. Her family were satisfied about the treatment and outcome.

## 3 Discussion

CEFB is defined as an impacted FB for at least one-week duration [4]. Miller et al. reported 8% of CEFB from a total 552 cases of FB ingestion [4]. They stated that the duration of impaction was less than 12 weeks in 83% of the patients and only 2 patients had a duration longer than two weeks [4]. Glover and colleagues presented a case of a child with a 2-year history of chronic cough after witnessed FB ingestion [3]. Also, in a case series of 5 children with FBs, Gilchrist et al. reported the retention duration from 2 to 24 months [5]. In addition, Cole et al. described 3 cases of prolonged FB retention from 3 to 12 months complicated by mediastinitis [7]. Moreover, Skaff et al. reported a case of 2-yeaar-old girl presented with intramural CEFB for 11 months [8]. To the best of our knowledge, we have presented a case for the longest duration of sharp FB impaction that presented after 26 months (approximately 113 weeks) as progressive dysphagia and solid food intolerance and revealed to have intramural “can tab” with technical perforation, esophageal stenosis, esophageal pouching and inflammatory mediastinal mass.

According to Miller et al., a ‘‘classic’’ esophageal perforation was defined as erosion through the mucosal and muscular layers of the esophagus such that there was a direct connection between the esophageal lumen and the mediastinum [4]. This was evaluated either by: (1) direct visualization of an esophageal perforation in the operating room (endoscopically or through an open approach) or (2) radiographically through visualization of esophageal contrast spilling into the mediastinum. A ‘‘technical’’ esophageal perforation is defined by meeting any one of the following criteria: (1) foreign body erosion through the mucosal layer of the esophagus; (2) a foreign body that had been walled off in either the mucosal or muscular layer of the esophagus; or (3) radiologic evidence of contrast leaking from the esophageal lumen into a loculation or space in the wall of the esophagus, but not extending into the mediastinum [4]. In their review, a total of 17 patients were found to have technical perforation. One of the main theoretical pathophysiology for development of CEFB and its complications is mucosal ulceration with prominent underlying necrosis of the muscularis propria followed by muscular parenchyma replacement by granulation and fibrous tissue, and a marked mixed inflammatory infiltrate extending into the surrounding tissue [4,9].

Recognition of the presenting symptoms of this condition is critical for the diagnosis and treatment of retained FBs. Incidence is greatest in children aged 6 months to 4 years. This reflects the tendency of small children to use their mouths in the exploration of their world [1,10]. When pediatric patients do present with CEFB, their symptoms differ from those of patients with acute FBs. Vomiting, dysphagia, drooling of saliva, and respiratory symptoms were the most common presenting symptoms in several studies [4,10,11]. In our case, the 36 months-patient presented with symptoms related to the complications of the FB ingestion-stricture formation-as progressive dysphagia and regurgitation for solid foods.

Most common FBs ingested in children are coins [4,11]. Being radiopaque hasten the process of diagnosis. Although the FB in our case was metallic, it was not visualized on neither her chest X-ray nor in the upper gastrointestinal series done for her. It is already reported that aluminum is a low molecular weight compound, relatively radiolucent, unlike most other common metals. Therefore, ingested aluminum objects are difficult to appreciate on radiographs [12,13]. The ingested FB in this case was Aluminum can tab with sharp edges.

In one study by Singh et al., the main factor that is associated with FB ingestion complication is the late presentation [14]. CEFB has been associated with severe complications including bronchoesophageal fistula, aortoesophageal fistula, esophageal perforation with resulting mediastinitis/abscess, complete esophageal stricture/esophageal obstruction, pulmonary edema, esophageal diverticulum, esophageal stricture requiring re-dilatation and malnutrition [[2], [3], [4], [5],^14^]. The main treatment modality is rigid esophagoscopy under general anesthesia. However, in severe cases, thoracotomy may be needed [4]. In this article, the patient underwent thoracotomy with removal of the FB and partial esophagectomy. The case was further complicated by severe stenotic tissue with pouching and intramural location of the FB.

Although the FB was removed, surgical site re-strictured again. Esophageal dilatation improved the condition of the patient temporarily. Unfortunately, she needed frequent dilatation. Mitomycin C is a popular choice for benign strictures [15]. Our case presents an example of how safe and effective Mitomycin C application in children with esophageal strictures requiring multiple frequent dilatations.

## 4 Conclusion

This report highlights and adds important learning points regarding esophageal FB ingestions in children. First, FB impaction or secondary stricture needs to be considered in the differential diagnosis of children presenting with new onset dysphagia and regurgitation. Second, metallic FBs might be even radiolucent, and X-ray might not be enough to exclude FB ingestion. Third, impacted FB in the esophagus may remain asymptomatic for some time and become symptomatic only when complications develop. Therefore, practitioners should keep a high index of suspicion for a retained esophageal FB in the child with gastrointestinal or respiratory symptoms that do not respond to standard therapy. Finally, CEFB can lead to significant esophageal injury and complications that may enforce the physicians to go for aggressive surgeries. As Esophageal dilatation would be considered a safe procedure in cases of esophageal strictures; Mitomycin C might be considered in resistant reoccurring strictures.

## Ethics approval

The institutional review board is not required.

## Sources of funding

No funding.

## Author contribution

All authors contributed significantly and in agreement with the content of the article. Tashtush, Al Quran and Aleshawi collected all data and photographs to draft the manuscript. Altamimi was the gastroenterologist who perform the esophagoscopy. Yusef was the pediatric consultant who follow the patient. Bataineh performed the surgery. Rousan and Khasawneh were the radiologist. Tashtush, Al Quran and Aleshawi wrote the manuscript for submission. All authors presented substantial contributions to the article and participated of correction and final approval of the version to be submitted.

## Conflicts of interest

The authors declare that they have no competing interests.

## Registration registration number

None.

## Guarantor

Dr. Eyad Altamimi.

## Consent for publication

The patient's parent gave their consent for publication of this Case report and any accompanying images. None of the images contains any patient's identifiers.

## Provenance and peer review

Not commissioned, externally peer reviewed.
